# Editorial: Shared Control for Tele-Operation Systems

**DOI:** 10.3389/frobt.2022.915187

**Published:** 2022-04-27

**Authors:** Yanan Li, Atsushi Takagi, Keng Peng Tee

**Affiliations:** ^1^ Department of Engineering and Design, University of Sussex, Brighton, United Kingdom; ^2^ Communication Science Laboratories, Nippon Telegraph and Telephone, Tokyo, Japan; ^3^ Institute for Infocomm Research, Agency for Science, Technology and Research, Singapore, Singapore

**Keywords:** shared control, tele-operation, physical human-robot interaction, human-robot collaboration, robot control

Tele-operation systems have been extensively studied in the literature and used in various robotic applications, such as in surgery, search and rescue, space exploration, nuclear decommissioning, etc. However, most traditional tele-operation systems adopt a leader-follower paradigm, where the tele-operated robot follows the human operator’s guidance. This unilateral interaction does not leverage the robot’s autonomy and imposes cognitive and physical loads on the human operator. Conversely, if the robot’s strengths such as local sensing and accurate, fast execution capabilities are fully utilized, the human’s control effort can be reduced while simultaneously increasing task efficiency and performance. Therefore, shared control between a human and a robot is essential to develop an advanced teleoperation system. However, designing shared control for teleoperation systems raises many challenges. From the robot’s perspective, decision marking must be in place to address questions such as when the robot should follow the human, when it should not, and what to do if there are conflicts between the two. The designer must also be aware of how the human adapts to the robot’s behavior, and how the latter’s design should incorporate such human motor adaptation.

In this Research Topic, we have invited researchers and practitioners in relevant fields to discuss these challenging issues. Four articles have been collected, which provide interesting insights on this topic and present promising results.

Zolotas et al. introduce a virtual reality system to assist human users in manipulation tasks. Their system provides human users visualization of joint limit and environmental constraints of the robot follower, by displaying manipulability polytopes at the teleoperated robot’s end-effector. They first use a pilot study to find graphical cues and virtual reality setup that are suitable for the task of screwing in a set of bolts. Then, through comparative experiments, they conclude that their system increases safety in terms of preventing erratic motion, despite reducing the task completion speed, compared to teleoperation without shared control.

Costi et al. investigate how shared control can mitigate the negative effects of time delay that is inevitable in teleoperation. They propose and compare four different control modalities of increasing autonomy: non-predictive human control (HC), predictive human control (PHC), (shared) predictive human-robot control (PHRC), and predictive robot control (PRC). They consider an object reaching and recognition task, and develop an internal model to predict the sensor’s output that is used to increase the robot’s autonomy. Their experimental results show that the two control architectures with increasing autonomy, PHRC and PRC, outperform the other two in terms of faster task completion and increased performance. By further comparing PHRC and PRC, they show that PHRC can avoid undesired hard contact with the environment that is observed under PRC, thus suggesting the advantage of shared control and concluding that PHRC represents a good trade-off between reaching accuracy, task completion speed and contact safety.

Hagmann et al. confirm the usefulness of virtual fixtures in teleoperation in minimally invasive robotic surgery, while they propose a shared control parametrization engine to retrieve procedural context information from a digital twin. Their idea is to abstract surgical training from real surgical operations, which offers a relatively well-defined environment and is thus easier to model. They first use a digital twin to estimate geometric and semantic task states, and then based on the estimation they parameterize assistance functions that are used to provide assistance in surgical training. Results from a pilot study demonstrate that novel users profit from haptic augmentation during training of certain tasks, such that they perform a task faster, more accurately and with less cognitive load.

Hu et al. take an overview of shared control for teleoperation, which they refer to as human–machine telecollaboration paradigm in their opinion article. They separate the telecollaboration paradigm into three sub-frameworks: human–machine bidirectional augmentation framework, where human and machine contribute to a same task with flexible roles; augmented machine intelligence under human guidance, where the machine infers the human’s intent to enhance its intelligence; augmented human performance leveraging machine intelligence, where the machine provides assistance to the human. The authors particularly review the deployment of large-scale autonomous robots during the Covid-19 pandemic and show the feasibility and great potential of telecollaboration.

